# Predictor‐Based Output Feedback Control of Tumour Growth With Positive Input: Application to Antiangiogenic Therapy

**DOI:** 10.1049/syb2.70005

**Published:** 2025-04-24

**Authors:** Mohamadreza Homayounzade

**Affiliations:** ^1^ Department of Mechanical Engineering Fasa University Fasa Iran

**Keywords:** antiangiogenic therapy, output feedback control, positive input, stability analysis, time‐delay systems, tumour growth control

## Abstract

Controlling tumour growth systems presents significant challenges due to the inherent restriction of positive input in biological systems, along with delays in system output and input measurements. Traditional control methods struggle to address these issues effectively, as they rely heavily on real‐time feedback from system outputs. The delays in output measurements can lead to instability in closed‐loop systems, whereas the inability of conventional approaches to manage the positive input constraint often results in ineffective control. In this study, the authors propose a novel control system designed to overcome these challenges. First, a system state prediction observer that utilises delayed output measurements was developed. Next, a backstepping technique was utilized to develop a feedback controller that ensures the control input stays positive, thereby guaranteeing the system's asymptotic stability. Furthermore, numerical comparisons with previous research validate the effectiveness of the proposed strategy. Overall, the approach offers a promising solution to the issues of delays and positive input constraints in tumour growth control systems.

## Graphical abstract

This study focuses on time delays in both input (inhibitor administration) and output (tumour growth measurement) in tumour growth control, proposing an observer that uses delayed output data to predict system states, while considering both input and output delays and system positivity for the first time. This paper introduces a novel method for directly controlling a system's actual input, avoiding the need for a virtual input or system extension used in previous studies, thereby reducing the risk of the input becoming unbounded and enabling more precise input management.

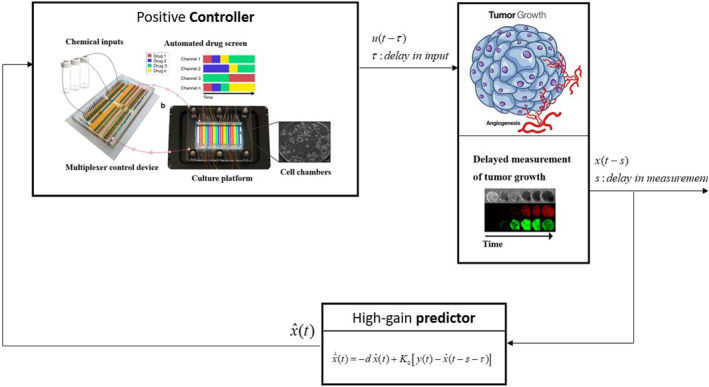

## 1 Introduction

Designing controllers for systems with positive input constraints presents significant challenges in control theory [1]. Positive systems, characterised by their nonnegative input requirements, are commonly encountered in fields such as blood glucose regulation [2, 3, 4], and tumour growth modelling [5, 6, 7, 8]. These systems demand specialised control strategies to ensure stability and performance, given their inherent input limitations.

A key challenge in designing controllers for tumour growth systems is the absence of real‐time tumour growth measurements, leading to delays in both system monitoring and inhibitor administration [8]. Consequently, controlling such a system involves managing a nonlinear system with delays in both input and output, a particularly difficult task in control science. Time delays can destabilise closed‐loop systems and reduce the effectiveness of control measures [9]. The primary limitation of these controllers is their inability to provide rapid and precise feedback, which can result in issues such as oscillations, instability, and diminished performance. To overcome these challenges, various strategies have been proposed, including robust control techniques, adaptive control, and predictive control, all aimed at improving system stability and effectiveness despite the complexities introduced by time delays and system nonlinearities.

In the output feedback control of time‐delay systems, state observers are frequently used to estimate unmeasured states [10, 11]. However, designing observers for nonlinear time‐delay systems poses significant challenges.

In ref. [11], Germani et al. applied the Grönwall lemma to derive sufficient conditions on delays that guarantee the exponential convergence of estimation errors in a chain observer. These conditions were subsequently relaxed in ref. [12] by Kazantzis and Wright. In ref. [13], Ahmed‐Ali et al. introduced a Lyapunov–Krasovskii functional, establishing a relationship between delay and the number of cascade observers with specific vector gains. This methodology has been further developed in ref. [14] by Ahmed‐Ali et al., where the focus is on proving the exponential convergence of observer estimation errors.

More recently, in refs. [10, 15], the exponential stability of closed‐loop systems has been demonstrated under the assumption of no disturbances. Predictor techniques, such as those discussed in ref. [15] by Karafyllis et al., have shown applicability to time delays that are not necessarily small, provided accurate system models are available. Although high‐gain observers are effective in mitigating uncertain nonlinearities, they restrict permissible delays to small values. However, they can still successfully recover the performance of state feedback control.

In ref. [16], Lei and Khalil proposed a high‐gain predictor‐based approach for a class of nonlinear systems, compensating for time delays to achieve robust and precise output feedback control. Additionally, robust control strategies for nonlinear systems have been explored in refs. [17, 18, 19] to ensure stability and performance despite the presence of input delays.

Previous research on control strategies for physiological systems has largely concentrated on achieving robustness against parameter uncertainties [20, 21]. However, the requirement for positive system inputs is frequently overlooked in controller design. The widespread occurrence of systems requiring positive inputs poses significant challenges that can impede the development of effective control strategies. These systems demand not only the consistent maintenance of positive input values but also must contend with the complexities of parameter uncertainties and nonlinearities, which are typical in many biological processes.

Previous efforts to address the positive input constraint in controller design have employed various strategies. One common approach has been to apply positive system inputs by saturating the controller output, as demonstrated in ref. [22]. Other strategies involve extending system models and incorporating virtual inputs [23, 24, 25, 26, 27]. Although these methods effectively ensure positive inputs, they come with significant drawbacks. Extending system models can increase complexity and may result in potentially unbounded inputs. Additionally, traditional controllers are primarily concerned with ensuring boundedness of system errors. Furthermore, previous approaches have typically relied on simulation‐based robustness assessments rather than formal mathematical justifications. These methods typically introduce a bilinear differential equation that enhances the system model by incorporating a virtual input, thereby establishing a direct connection between the real and virtual inputs. This equation ensures that the real input remains positive for all values of the virtual input, effectively shifting the challenge to regulating the virtual input while maintaining a positive real input to control the extended system. However, despite these advancements, challenges persist, particularly the nonlinearity introduced by extending the system as discussed in ref. [25]. Additionally, several existing approaches, including linear parameter variation (LPV) methods [27], feedback linearisation techniques [25], and robust norm‐based controllers [26], have significant limitations.

In ref. [8], the control of tumour growth using positive inputs is examined. To address the limitations of existing methods, the research proposes a novel control framework that directly regulates the system's actual input, ensuring the positivity of inputs without needing system extension. However, the proposed strategy depends on real‐time monitoring of tumour volume, without considering potential delays in measurement and injection administration.

In this paper, we aim to address the limitations identified in ref. [8] by developing an observer that estimates system states using delayed measurements. Furthermore, Lyapunov's theorem is employed to rigorously evaluate the robustness of the proposed controller in the face of system uncertainties and input delays. A key advantage of our controller lies in its simplicity, requiring only tumour volume measurements, unlike approaches that depend on additional sensors or estimation filters. This streamlined control strategy offers an efficient and reliable solution for managing tumour growth systems, effectively overcoming critical shortcomings of prior methods. The distinct advantages of the proposed controller, when compared to earlier methods, can be highlighted as follows:

1. This study investigates the time delays in both input and output within the system, with a particular emphasis on delays related to inhibitor administration and tumour growth measurement in the context of tumour growth control. Distinct from prior research, we propose an observer that leverages time‐delayed output data to predict the system's states. To the best of our knowledge, this is the first instance where the delays in both measurements and system input, alongside the system's positivity, are simultaneously taken into account.
2. In previous research [23, 24, 25, 26, 27], ensuring the system's positivity involved extending the actual system by incorporating a bilinear differential equation. This equation created a direct relationship between the virtual input (v) and the real input (u), such that $\dot{u}=-\mathrm{uv}$. Subsequently, using $u=u\left(0\right)\;\exp\left(-\int_{0}^{t}v\left(\tau \right)d\tau \right)$, the real input u was determined, ensuring a positive actual input for every magnitude of the virtual input v(t). Without direct control over the real input, the value of u(t) could increase over time for specific values of v(t), particularly if the virtual input's time history included negative values. In contrast, this paper presents a novel approach that directly controls the system's actual input, removing the need for a virtual input or system model extension. This method effectively minimises the risk of the input becoming unbounded, a drawback identified in previous studies [23, 24, 25, 26, 27], and enables more precise management of the actual input.
3. In previous studies [25, 26, 27], controllers were primarily designed to ensure $H_{\infty }$ and/or $H_{2}$ system stability, with a focus on maintaining the boundedness of system states. In contrast, this paper adopts a more thorough approach to stability analysis by not only examining the equilibrium point but also developing a robust controller that guarantees asymptotic stability for the tumour growth model. Unlike prior methods, our controller design does not require extending the system model. Instead, we utilise the backstepping (BS) control technique, a recursive feedback method that stabilises the system's origin, offering precise control over each state and significantly enhancing the overall effectiveness of the control strategy.
4. Previous studies [22, 23, 24, 25, 26] primarily relied on simulation experiments to evaluate system robustness. In contrast, this paper adopts a more rigorous approach by employing Lyapunov theory and thorough mathematical analysis to assess the system's robustness. Our analysis shows that the controlled system ensures bounded input‐bounded output (BIBO) stability, even in the presence of disturbances. Additionally, the robustness of the proposed controller is thoroughly validated through extensive simulations as detailed in Section 5. By varying tumour growth parameters such as treatment efficacy and tumour cell proliferation rates by 10%–100%, the simulations convincingly show that the closed‐loop system remains resilient to parametric uncertainties while maintaining positive input, thereby underscoring the effectiveness of our control method.
5. In previous studies [25, 26], control gain design often involved iterative methods to minimise the norm of the closed‐loop system, while [27] utilised the LPV method to develop a parameter‐dependent feedback gain matrix. These approaches, however, are frequently hindered by their complexity. By contrast, the control structure proposed in this study provides a much simpler and more efficient alternative to those in refs. [25, 26, 27], leading to a more streamlined and effective controller implementation.
6. In earlier research [25, 26], controllers relied on measurements of both inhibitor levels and tumour volume. Additionally, studies [23, 24] required knowledge of the inhibitor injection rate, along with tumour volume and inhibitor levels. To overcome these challenges [27], introduced a stabilising discrete Kalman filter designed to estimate the inhibitor level, thus eliminating the need for direct measurement in the control law. As highlighted in ref. [28], analysing the stability of the observer and controller independently can lead to instability in the closed‐loop system under certain initial conditions.

The structure of the following sections is outlined as follows: Section 2 introduces the state‐space system modelling, details the controller design using the BS approach, and presents the error systems. Section 3 focuses on the design of the high‐gain predictor for tumour volume estimation. In Section 4, we examine the stability of the system through the application of the Lyapunov theorem. Section 5 is dedicated to validating our theoretical results with numerical simulations, providing a rigorous comparison with the findings from [25, 26, 27]. Finally, Section 6 concludes the paper, summarising the key insights.

***Notation:*** *Throughout this article* |.| *denotes the absolute value, while* I *represents the identity matrix. The symbol* λ *refers to eigenvalues, with* $\lambda_{\min}\left(\cdot \right)$ *and* $\lambda_{\max}\left(\cdot \right)$ *indicating the maximum and minimum eigenvalues of a matrix, respectively. The superscript ‘T’ denotes the transpose of a matrix. The notation* $R^{n}$ *represents the n‐dimensional Euclidean space, and* ‖.‖ *indicates the Euclidean norm. The set* $R^{+}$ *refers to non‐negative real numbers. For a positive constant* $r\in R^{+}$ *denotes the value of* x(t) *at time* t *, and* $x_{t}$ *is an element of* $C^{n}$ *defined by* $x_{t}\left(\alpha \right)=x\left(t+\alpha \right)$, −r≤α≤0 *with the norm* ${\left\|x_{t}\right\|}_{s}=\sup_{\theta \in \left[-r,0\right]}\left\|x\left(t+\alpha \right)\right\|\leq \beta$ *, where* β *is a positive constant. The variable* $x\left(t\text{;}\;t_{0},\varphi \right)$ *represents the solution of the time‐delay system with initial time* $t_{0}$ *and initial condition* φ *. Finally, given a Lyapunov functional* $V\left(x_{t},{\dot{x}}_{t},t\right)\text{:}C^{n}\times L_{2}\times R\to R^{+}$ *, we have* $\dot{V}\left(x_{t},{\dot{x}}_{t},t\right)=\lim_{q\to 0^{+}}\;\sup\frac{1}{q}\left[V\left(x_{t+h},{\dot{x}}_{t+h},t+h\right)-V\left(x_{t},{\dot{x}}_{t},t\right)\right]$.

## 2 Tumour Growth Modelling

In this section, we start by presenting the tumour growth model in state‐space form. We then outline the procedure for designing the controller and proceed to describe the associated error systems.

### 2.1 System Modelling

Based on research conducted in refs. [23, 24, 25, 26, 27], tumour growth can be expressed in state‐space form as follows:

(1a) $$\frac{dx\left(t\right)}{dt}=\text{ax}\left(t\right)-\mathrm{bx}\left(t\right)\;z\left(t\right),$$

(1b) $$\frac{\text{dz}\left(t\right)}{\text{dt}}=-\text{cz}\left(t\right)+u\left(t-\tau \right),$$

(1c) y(t)=x(t−s),

In this model, the state x∈R represents the tumour volume over time in $mm^{3}$, while z∈R denotes the time‐dependent inhibitor level in mg/kg. The variable u describes the inhibitor injection rate over time in mg/kg/day, and y∈R is the system output. The parameters τ and s represent sufficiently small input and output delays, respectively, with τ,s∈[0,r], where r=τ+s. Parameter a indicates the tumour proliferation rate in 1/day, reflecting the rate of tumour cell division, while parameter b represents the drug's inhibition effect in kg/mg/day, which measures the drug's efficacy. Additionally, c denotes the drug clearance rate in 1/day, indicating how quickly the drug is depleted from the system.

### 2.2 Controller Design Methodology

Let us define δ(t)≜t+τ, consequently we have the following:

(2a) $$\begin{array}{l} \dot{x}\left(\delta \left(t\right)\right)=\frac{\mathrm{dx}\left(\delta \left(t\right)\right)}{\mathrm{dt}}=\frac{\text{dx}\left(\delta \left(t\right)\right)}{d\delta \left(t\right)}\frac{d\delta \left(t\right)}{\text{dt}}=\frac{\text{dx}\left(\delta \left(t\right)\right)}{d\delta \left(t\right)}=a\;x\left(\delta \left(t\right)\right)-b\;x\left(\delta \left(t\right)\right)\;z\left(\delta \left(t\right)\right), \end{array}$$

(2b) $$\dot{z}\left(\delta \left(t\right)\right)=\frac{\text{dz}\left(\delta \left(t\right)\right)}{\text{dt}}=\frac{\text{dz}\left(\delta \left(t\right)\right)}{d\delta \left(t\right)}\frac{d\delta \left(t\right)}{\text{dt}}=\frac{\text{dz}\left(\delta \left(t\right)\right)}{d\delta \left(t\right)}=-c\;z\left(\delta \left(t\right)\right)+u\left(t\right).$$

In this section, we focus on designing the control input u(t), under the assumption that the state x(δ(t)) is fully accessible for feedback. Subsequently, in Section 3, we proceed to develop an observer designed to estimate the system state, denoted as $\hat{x}\left(t\right)$, which will be used as a substitute for the actual state x(δ(t)) in the control law.

Let's define the variable $e_{z}$ as follows:

(3) $$e_{z}=z\left(\delta \right)-z^{*}.$$

When Equation (2a) is combined with the desired magnitude of z, denoted by $z^{*}$ and carefully selected in subsection C using the BS technique, the formula (2a) can be reformulated as follows:

(4) $$\begin{array}{l} \dot{x}=ax-bx\left(z-z^{*}+z^{*}\right),\\ =ax-bx\left(e_{z}+z^{*}\right). \end{array}$$

Therefore, $z^{*}$ can serve as a control variable to manage the state x. In subsection 3, the desired value of $z^{*}$ is carefully determined using the BS technique, ensuring that as z approaches $z^{*}$, the state x asymptotically converges to zero.

Differentiating definition (3) yields the following:

(5) $${\dot{e}}_{z}=\dot{z}-{\dot{z}}^{*}.$$

Upon substitution of Equation (2b) into the obtained result, we arrive at the following:

(6) $${\dot{e}}_{z}=-cz+u\left(t\right)-{\dot{z}}^{*}.$$

Therefore, the control input must be configured to ensure that z approaches $z^{*}$, thereby causing the state $e_{z}$ to converge to zero.

The backstepping methodology offers a recursive approach for stabilising the equilibrium point of systems expressed in strict‐feedback form. The control input u, crafted using backstepping, provides the most direct stabilising effect on the state z, as illustrated by systems (2a) and (2b). Examining Equation (4) highlights the independent function of the state $z^{*}$ as a stabilising control for x. Consequently, $z^{*}$ is precisely calibrated to ensure the stabilisation of the state x.

By utilising $z^{*}$ and examining the governing equation (Equation 4), the backstepping method effectively stabilises the x subsystem. This approach is then extended in Equation (6) to formulate the control law u, guaranteeing that $e_{z}$ converges to zero, or equivalently, $\lim_{t\to \infty }z=z^{*}$.

The controller methodology, as outlined through the BS technique is described as follows:

1. To ensure that the state x converges exponentially to zero as z approaches $z^{*}$, the variable $z^{*}$ is precisely designed
2. The control input u is carefully designed to guarantee that the tracking error $e_{z}$ converges to zero exponentially, ensuring that z approaches the desired trajectory $z^{*}$.

Let's look at output state feedback control that has the following structure u(t)=γ(x(t+τ)), such that u(t−τ)=γ(x(t)), where assumption one is met by the function γ(·). In Section 3, measurements of the system output are used to develop an observer that predicts the state x(δ(t)). This estimate, represented as $\hat{x}\left(t\right)$, is then entered into the control law, that is $u\left(t\right)=\gamma \left(\hat{x}\left(t\right)\right)$.

Assumption 1 The function γ(·) is locally Lipschitz in x. In Section 2, γ(·) is meticulously crafted to guarantee the exponential stability of the system. This design demonstrates the existence of a Lipschitz functional $V_{1}\left(x\right)$ and functions $\alpha_{1},\alpha_{2},\alpha_{3}$ of class κ such that

(7) $$\begin{array}{c} \alpha_{1}\left(X\right)\leq V_{1}\left(X\right)\leq \alpha_{2}\left(X\right)\;\\ {\dot{V}}_{1}\left(X\right)\leq -\alpha_{3}\left(X\right) \end{array}$$

*In this context, the augmented state* X *is characterised as*

(8) $$X=\left[\begin{array}{l} x\\ e_{z} \end{array}\right].$$

Remark 1 In order to reduce the chance of the system peaking during the transient response of the observer, we utilise saturated output feedback control as recommended in [28, 29]:

(9) $$u\left(t\right)=Y\;sat\left[\gamma \left(\hat{x}\left(t\right)\right)/Y\right]=\gamma_{s}\left(\hat{x}\left(t\right)\right)$$

*where* $Y\geq \max_{x\in \Omega_{c}}\left|\gamma \left(x\right)\right|$.

### 2.3 Controller Design

The desired magnitude, $z^{*}$, is formed using the BS technique as follows:

(10) $$z^{*}=k.$$

The positive control gain is indicated by the constant k in this instance.

Substituting Equation (10) for $z^{*}$ in Equation (4) yields the following result:

(11) $$\dot{x}=\left(a-bk\right)\;x-bxe_{z}.$$

Thus, when the control gain is configured appropriately,

(12) $$k>\frac{a}{b},$$

We have

(13) $$\dot{x}=-dx-bxe_{z},$$

where d=bk−a denotes a positive constant.

Using u(t)=γ(x(t+τ)), we can determine the control input. The value of γ(·) comes from the following equation:

(14) $$\gamma \left(x\left(t+\tau \right)\right)=cz^{*}+b{\left(x\left(t+\tau \right)\right)}^{2},$$

where $z^{*}$ is already defined by Equation (10).

**FIGURE 1:**
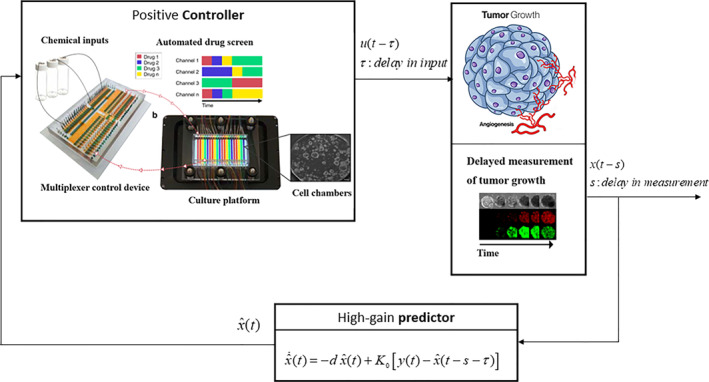
Schematic block diagram illustrating the predictor‐based control system.

Remark 2 Figure 1 illustrates a closed‐loop controlled system for tumour growth using a programmable microfluidic platform. The system employs a predictor to estimate tumour growth rates, which then serves as feedback for the controller. An automated microfluidic chip, featuring a membrane‐valve‐based multiplexer control device, enables dynamic stimulation profiles for a 3D cellular and organoid culture platform. The cross‐section of 3D culture chamber device highlights the ability to apply combinatorial and time‐varying chemical stimulations. Time‐lapse imaging facilitates continuous observation and quantification of organoid or 3D cellular structures, with the ability to change fluidic conditions on demand and disassemble for further cellular assays.

Remark 3 It is apparent from Equation (10) that $z^{*}$ is a positive signal. Additionally, the control input u(t), as specified by Equation (14), remains positive at all times because the term $bx^{2}$ is perpetually nonnegative.

Remark 4 It is noteworthy that the proposed controller only necessitates the measurement of the state x for feedback. In contrast, the controllers discussed in refs. [25, 26] require measurements of both states x and z.

Using Equation (14) as a substitute for the control input in the error system provided by Equation (6) and keeping in mind that ${\dot{z}}^{*}=0$, we obtain the following:

(15) $$\begin{array}{l} {\dot{e}}_{z}=-c\left(z-z^{*}\right)+bx^{2},\\ =-ce_{z}+bx^{2}. \end{array}$$

Theorem 1 *Equation* (7) *clearly illustrates that the stability of the system, ensured by the control input defined in Equation* (14), *fully satisfies the criteria for the Lyapunov function* $V_{1}$.

Select the subsequent Lyapunov function candidate:

(16) $$V_{1}=\frac{1}{2}x^{2}+\frac{1}{2}{e_{z}}^{2}.$$

Calculating the time derivative of Equation (16) produces the following:

(17) $${\dot{V}}_{1}=x\;\dot{x}+e_{z}\;{\dot{e}}_{z}.$$

Using Equation (17) in lieu of Equations (13) and (15) yields:

(18) $${\dot{V}}_{1}=x\left(-dx-bxe_{z}\right)+e_{z}\left(-ce_{z}+bx^{2}\right).$$

Simplification of Equation (18) produces the following:


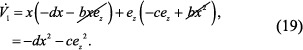




Consequently, we get

(20) $${\dot{V}}_{1}\leq -c_{3}\;V_{1}.$$

In this context, $c_{3}=2\;\min\left\{d,c\right\}$ denotes a positive constant. According to Equation (20), ${\dot{V}}_{1}\leq 0$.

Remark 5 The control input given by Equation (14) requires knowledge of x(t+τ) for feedback. However, due to a delay in the system output, only y(t)=x(t−s) is available for measurement. To address this, Section 3 introduces a predictor observer designed to estimate the state $\hat{x}\left(t\right)$, using the predictor state x(t+τ). This estimated state, $\hat{x}\left(t\right)$, will be used in the output feedback control, replacing the actual states x(t+τ) in Equation (14) for state feedback.

Theorem 2 *The Appendix demonstrates that the proposed controller maintains BIBO stability, even in the existence of uncertainties in the system parameters.*

## 3 Designing a High‐Gain Predictor

In order to replace the state x(t+τ) in Equation (14) with state feedback, we plan to use $\hat{x}\left(t\right)$ in the output feedback control.

Let us formulate the high‐gain predictor as follows:

(21) $$\dot{\hat{x}}\left(t\right)=-d\;\hat{x}\left(t\right)+K_{0}\;\left[y\left(t\right)-\hat{x}\left(t-s-\tau \right)\right].$$

Given the initial condition ${\hat{x}}_{0}$ satisfying ${\left|{\hat{x}}_{0}\right|}_{s}\leq \eta$, where η is a positive constant, the observer gain $K_{0}$ is determined by the following:

(22) $$K_{0}=\frac{a_{1}}{\varepsilon }.$$

Here, $a_{1}$ represents a positive constant, and ε denotes the predictor gain. Let's define δ(t)≜t+τ. According to system (13), the state x(δ(t)) is determined by the following:

(23) $$\dot{x}\left(\delta \left(t\right)\right)=\frac{dx\left(\delta \left(t\right)\right)}{d\delta \left(t\right)}=-d\;x\left(\delta \left(t\right)\right)-b\;x\left(\delta \left(t\right)\right)\;e_{z}\left(\delta \left(t\right)\right),$$

The predictor state $\hat{x}\left(t\right)$ provides an estimation of x(δ(t)) with improved precision. The behaviour of the estimation error $e\left(t\right)=x\left(\delta \left(t\right)\right)-\hat{x}\left(t\right)$ is determined by the following:

(24) $$\begin{array}{l} \dot{e}\left(t\right)=\dot{x}\left(\delta \left(t\right)\right)-\dot{\hat{x}}\left(t\right)\\ =-d\;x\left(\delta \left(t\right)\right)-b\;x\left(\delta \left(t\right)\right)e_{z}\left(\delta \left(t\right)\right)+d\;\hat{x}\left(t\right)-K_{0}\left[x\left(t-s\right)-\hat{x}\left(t-s-\tau \right)\right]. \end{array}$$

Therefore, we obtain the following:

(25) $$\dot{e}\left(t\right)=-d\left(x\left(\delta \left(t\right)\right)-\hat{x}\left(t\right)\right)+b\Delta_{2}\left(X\left(\delta \left(t\right)\right)\right)-K_{0}\;e\left(t-s-\tau \right),$$

where

(26) $$\Delta_{2}\left(\cdot \right)=-x\;e_{z}.$$

Consequently, we obtain the following:

(27) $$\dot{e}\left(t\right)=-d\;e\left(t\right)-K_{0}\;e\left(t-s-\tau \right)+b\Delta_{2}\left(X\left(\delta \left(t\right)\right)\right).$$

Considering that

(28) $$e\left(t-s-\tau \right)=e\left(t\right)-\int_{t-s-\tau }^{t}\dot{e}\left(\omega \right)\;d\omega ,$$

The prediction error system is characterised by the following:

(29) $$\varepsilon \dot{e}\left(t\right)=A\;e\left(t\right)-\varepsilon K_{0}\;e\left(t\right)+\varepsilon K_{0}\;J+\varepsilon b\;\Delta_{2}\left(\cdot \right).$$

here, ε represents the predictor gain, with A=−εd, and

(30) $$J=\int_{t-s-\tau }^{t}\dot{e}\left(\omega \right)\;d\omega .$$

By assigning $A_{0}=A-\varepsilon K_{0}$, Equation (29) can be restructured as follows:

(31) $$\varepsilon \dot{e}\left(t\right)=A_{0}\;e\left(t\right)+\varepsilon K_{0}\;J+\varepsilon b\;\Delta_{2}\left(\cdot \right).$$

It is important to recognise that $\hat{x}\left(t\right)$ denotes the predicted state of x(δ(t)). In the absence of perturbations, and based on Equation (31), we derive the following:

(32) $$\varepsilon \dot{e}\left(t\right)=Ae\left(t\right)-\varepsilon K_{0}e\left(t-s-\tau \right)=A_{0}\;e\left(t\right)+\varepsilon K_{0}\;J.$$

Section 2 demonstrates that applying the input u(t)=γ(x(t+τ)), with γ(·) defined by Equation (14), results in the closed‐loop system given by the following:

(33) $$\dot{X}\left(t\right)=f\left(X\left(t\right)\right),$$

where

(34) $$f\left(\cdot \right)=\left[\begin{array}{l} -dx-bxe_{z}\\ -ce_{z}+bx^{2} \end{array}\right]$$

Section 2 demonstrates that the equilibrium point of the system $\dot{X}\left(t\right)=f\left(X\left(t\right)\right)$ exhibits exponential stability.

Using the bounded output feedback control $u\left(t\right)=\gamma \left(\hat{x}\left(t\right)\right)$, with γ(·) defined according to Equation (14), namely:

(35) $$\gamma \left(\hat{x}\left(t\right)\right)=cz^{*}+b{\left(\hat{x}\left(t\right)\right)}^{2}.$$

Consequently, the closed‐loop system can be represented as follows:

(36) $$\dot{X}\left(t\right)=F\left(X\left(t\right),0\right)-F\left(X\left(t\right),0\right)+F\left(X\left(t\right),e\right).$$

Here, F(X(t),0)=f(X(t)) represents the closed‐loop system driven by the input u(t)=γ(x(t+τ)). In Section 3, an observer is developed to predict the state x(t+τ) based on the system's output measurements. The estimated state, $\hat{x}\left(t\right)$, is then incorporated into the control law $u\left(t\right)=\gamma \left(\hat{x}\left(t\right)\right)$. Substituting the estimated state $\hat{x}\left(t\right)$ for x(t+τ) can be viewed as a perturbation, that is,

(37) $$\Delta_{1}\left(\cdot \right)=F\left(X\left(t\right),e\right)-F\left(X\left(t\right),0\right).$$

By substituting Equation (37) into Equation (36) and incorporating Equation (31), the closed‐loop system is derived as follows:

(38a) $$\dot{X}\left(t\right)=f\left(X\left(t\right)\right)+B\;\Delta_{1}\left(x,e\right),$$

(38b) $$\varepsilon \;\dot{e}\left(t\right)=A_{0}\;e\left(t\right)+\varepsilon \;K_{0}\;J+\varepsilon \;b\Delta_{2},$$

where $B=\left[\begin{array}{l} 0\\ 1 \end{array}\right]$. The term $\Delta_{2}$, as defined previously by Equation (26), denotes the perturbation. It can be confirmed that $\Delta_{1}\left(\cdot \right)$ and $\Delta_{2}\left(\cdot \right)$ are locally Lipschitz in (x,e). Additionally, $\Delta_{1}\left(0,0\right)=0$, $\Delta_{2}\left(0,0\right)=0$, and within the region $\Omega_{d}=\left\{X\left(t\right)|\;\left\|X\left(t\right)\right\|\leq l_{d}\right\}$, we have the following:

(39) $$\left\|\Delta_{1}\left(\cdot \right)\right\|\leq l_{1}\;{\left|e_{t}\right|}_{s},\left|\Delta_{2}\left(\cdot \right)\right|\leq \beta {\left|e_{t}\right|}_{s}$$

Here, $l_{1},\beta$ represent positive constants.

Theorem 3 *Section* 4 *demonstrates that the proposed controller, in conjunction with the state‐predicting observer, ensures the asymptotic stability of the equilibrium point.*

## 4 Analysis of Stability

Let us establish the Lyapunov function as follows:

(40) $$W=V_{1}+V_{2},$$

where

(41) $$V_{1}=\frac{1}{2}X^{T}X,$$

(42) $$V_{2}=Pe^{2}+\int_{t-r}^{t}\left(\omega -t+r\right){\left|\dot{e}\left(\omega \right)\right|}^{2}d\omega ,$$

and

(43) $$P=\frac{1}{2A_{0}}.$$

It can be verified that $\left|V_{2}\left(t,e_{t}^{\prime \prime }\right)-V\left(t,e_{t}^{\prime }\right)\right|\leq c_{4}\left({\left|e_{t}^{\prime \prime }\right|}_{s}+{\left|e_{t}^{\prime }\right|}_{s}\right)\left|e_{t}^{\prime \prime }-e_{t}^{\prime }\right|$.

Analysing the system with prediction according to Equation (38a) and applying the same methodology outlined in Section 2, the derivative of $V_{1}\left(X\left(t\right)\right)$ with respect to (38a), which satisfies property (7), is given by the following:

(44) $$\begin{array}{l} {\dot{V}}_{1}\leq -c_{3}{\left\|X\left(t\right)\right\|}^{2}+\left\|X\left(t\right)\right\|\;\left|\Delta_{1}\left(\cdot \right)\right|\\ \leq -c_{3}{\left\|X\left(t\right)\right\|}^{2}+l_{1}\left\|X\left(t\right)\right\|\;{\left|e_{t}\right|}_{s}. \end{array}$$

Here, $c_{3}=2\;\min\left\{d,c\right\}$ represents a positive constant.

The derivative of $V_{2}\left(e_{t},{\dot{e}}_{t}\right)$ yields

(45) $$\begin{array}{l} {\dot{V}}_{2}=\lim_{h\to 0^{+}}\sup\frac{1}{h}\left[V_{2}\left(t+h,e_{t+h}^{\left(31\right)},\varepsilon \right)-V_{2}\left(t,e_{t},\varepsilon \right)\right]\\ =\lim_{h\to 0^{+}}\sup\frac{1}{h}\left[V_{2}\left(t+h,e_{t+h}^{\left(32\right)},\varepsilon \right)-V_{2}\left(t,e_{t},\varepsilon \right)\right]\\ +\lim_{h\to 0^{+}}\sup\frac{1}{h}\left[V_{2}\left(t+h,e_{t+h}^{\left(31\right)},\varepsilon \right)-V_{2}\left(t+h,e_{t+h}^{\left(32\right)},\varepsilon \right)\right]\\ ={{\dot{V}}_{2}|}_{\left(32\right)}+\lim_{h\to 0^{+}}\sup\frac{1}{h}\left[V_{2}\left(t+h,e_{t}+\frac{h}{\varepsilon }\left(A_{0}\;e_{t}+\varepsilon K_{0}\;J_{t}+\varepsilon b\;\Delta_{2}\left(\cdot \right)\right),\varepsilon \right)-V_{2}\left(t+h,e_{t}+\frac{h}{\varepsilon }\left(A_{0}\;e_{t}+\varepsilon K_{0}\;J_{t}\right),\varepsilon \right)\right]. \end{array}$$

In this context, ${{\dot{V}}_{2}|}_{\left(32\right)}$ represents the derivative of $V_{2}$ through the unperturbed system (32). The terms $e_{t+h}^{\left(31\right)}$ and $e_{t+h}^{\left(32\right)}$ denote the solutions of the perturbed system (31) and the unperturbed system (32), respectively, at time t+h, both originating from $e_{t}$ at time t. Additionally, $J_{t}=\int_{t+\theta -\left(\tau +s\right)}^{t+\theta }\dot{e}\left(\tau \right)\;d\tau$ is considered for θ∈[−r,0].

The time derivative of $V_{2}$ results in the following:

(46) $${\dot{V}}_{2}=2Pe\;\dot{e}+r{\left|\dot{e}\left(t\right)\right|}^{2}-\int_{t-r}^{t}{\left|\dot{e}\left(\omega \right)\right|}^{2}d\omega .$$

Upon calculating ${\dot{V}}_{2}$ along (32), we obtain the following:

(47) $${{\dot{V}}_{2}|}_{\left(32\right)}=\frac{1}{\varepsilon }2Pe\;\left(A_{0}\;e\left(t\right)+\varepsilon K_{0}\;J\right)+r{\left|\dot{e}\left(t\right)\right|}^{2}-\int_{t-r}^{t}{\left|\dot{e}\left(\omega \right)\right|}^{2}d\omega .$$

Simplification of Equation (47) results in the following:

(48) $${{\dot{V}}_{2}|}_{\left(32\right)}\leq -\frac{1}{\varepsilon }{\left|e\left(t\right)\right|}^{2}+\frac{2}{\varepsilon }\left|\varepsilon P\;K_{0}\right|\;\left|e\left(t\right)\right|\;\left|J\right|+r{\left|\dot{e}\left(t\right)\right|}^{2}-\int_{t-r}^{t}{\left|\dot{e}\left(\omega \right)\right|}^{2}d\omega .$$

Applying Young's inequality to Equation (32) yields

(49) $${\left|\dot{e}\left(t\right)\right|}^{2}\leq \frac{2{A_{0}}^{2}}{\varepsilon^{2}}{\left|e\left(t\right)\right|}^{2}+2{K_{0}}^{2}{\left|J\right|}^{2}.$$

Considering that for any arbitrary vectors $q_{1},q_{2}$ we have $-q_{1}^{2}+2q_{1}\;q_{2}=-{\left(q_{1}-q_{2}\right)}^{2}+q_{2}^{2}$, we obtain the following:

(50) $$-\frac{1}{2\varepsilon }{\left|e\left(t\right)\right|}^{2}+\frac{2}{\varepsilon }\left|\varepsilon PK_{0}\right|\;\left|e\left(t\right)\right|\;\left|J\right|=-\frac{1}{2\varepsilon }\left[{\left(\left|e\left(t\right)\right|-2\left|\varepsilon PK_{0}\right|\;\left|J\right|\right)}^{2}-4{\left|\varepsilon PK_{0}\right|}^{2}\;{\left|J\right|}^{2}\right]\leq \frac{2}{\varepsilon }{\left|\varepsilon PK_{0}\right|}^{2}\;{\left|J\right|}^{2}.$$

Utilising Jensen's inequality ${\left|J\right|}^{2}\leq r\int_{t-r}^{t}{\left|\dot{e}\left(\omega \right)\right|}^{2}d\omega$ we obtain the following:

(51) $$-\frac{1}{2\varepsilon }{\left|e\left(t\right)\right|}^{2}+\frac{2}{\varepsilon }\left|\varepsilon PK_{0}\right|\;\left|e\left(t\right)\right|\;\left|J\right|\leq \frac{2r}{\varepsilon }{\left|\varepsilon PK_{0}\right|}^{2}\;\int_{t-r}^{t}{\left|\dot{e}\left(\omega \right)\right|}^{2}d\omega .$$

Replacing Equations (49) and (51) into Equation (48) yields

(52) $$\begin{array}{l} {{\dot{V}}_{2}|}_{\left(32\right)}\leq -\frac{1}{4\varepsilon }{\left|e\left(t\right)\right|}^{2}-\left[\frac{1}{4\varepsilon }-\frac{2r{A_{0}}^{2}}{\varepsilon^{2}}\right]{\left|e\left(t\right)\right|}^{2}-\left[1-\frac{2{\left|\varepsilon PK_{0}\right|}^{2}r}{\varepsilon }-\frac{2{\left|\varepsilon K_{0}\right|}^{2}r^{2}}{\varepsilon^{2}}\right]\;\int_{t-r}^{t}{\left|\dot{e}\left(\omega \right)\right|}^{2}d\omega \\ \leq -\frac{1}{4\varepsilon }{\left|e\left(t\right)\right|}^{2}-\frac{1}{2r}r\;\int_{t-r}^{t}{\left|\dot{e}\left(\omega \right)\right|}^{2}d\omega -\left[\frac{1}{4\varepsilon }-\frac{2r{A_{0}}^{2}}{\varepsilon^{2}}\right]{\left|e\left(t\right)\right|}^{2}-\left[\frac{1}{2}-\frac{2{\left|\varepsilon PK_{0}\right|}^{2}\;r}{\varepsilon }-\frac{2{\left|\varepsilon K_{0}\right|}^{2}\;r^{2}}{\varepsilon^{2}}\right]\;\int_{t-r}^{t}{\left|\dot{e}\left(\omega \right)\right|}^{2}d\omega . \end{array}$$

Choosing

(53) $$\begin{array}{l} \frac{1}{4\varepsilon }-\frac{2r{A_{0}}^{2}}{\varepsilon^{2}}>0\;\Rightarrow \;\varepsilon >8r{A_{0}}^{2},\\ \frac{2{\left(\varepsilon PK_{0}\right)}^{2}\;r}{\varepsilon }<\frac{1}{4}\;\Rightarrow \;\varepsilon >8r{\left(Pa_{1}\right)}^{2},\\ \frac{2{\left(\varepsilon K_{0}\right)}^{2}\;r^{2}}{\varepsilon^{2}}<\frac{1}{4}\;\Rightarrow \;\varepsilon >\sqrt{8}\;a_{1}r. \end{array}$$

In other words, lets choose ε≥σr where

(54) $$\sigma =\max\left\{8{A_{0}}^{2},8{\left(Pa_{1}\right)}^{2},\sqrt{8}\;a_{1}\right\}$$

Consequently, the coefficients of the last two terms in Equation (52) are non‐negative. This is due to the fact that

(55) $$r\int_{t-r}^{t}{\left|\dot{e}\left(\omega \right)\right|}^{2}d\omega \geq \int_{t-r}^{t}\left(\omega -t+r\right){\left|\dot{e}\left(\omega \right)\right|}^{2}d\omega .$$

The inequality (52) can be interpreted as follows:

(56) $$\begin{array}{l} {{\dot{V}}_{2}|}_{\left(32\right)}\leq -\frac{1}{4\varepsilon }{\left|e\left(t\right)\right|}^{2}-\frac{1}{2r}\int_{t-r}^{t}\left(\omega -t+r\right){\left|\dot{e}\left(\omega \right)\right|}^{2}d\omega \\ \leq -\frac{1}{\varepsilon }\left[\frac{P{\left|e\left(t\right)\right|}^{2}}{4\left|P\right|}+\frac{\sigma }{2}\int_{t-r}^{t}\left(\omega -t+r\right){\left|\dot{e}\left(\omega \right)\right|}^{2}d\omega \right]\\ \leq -\frac{1}{\varepsilon }\min\left\{\frac{1}{4\left|P\right|},\frac{\sigma }{2}\right\}\left[P{\left|e\left(t\right)\right|}^{2}+\int_{t-r}^{t}\left(\omega -t+r\right){\left|\dot{e}\left(\omega \right)\right|}^{2}d\omega \right]\\ \leq -\frac{{\tilde{c}}_{3}}{\varepsilon }V_{2}, \end{array}$$

where ${\tilde{c}}_{3}=\min\left\{\frac{1}{4\left|P\right|},\frac{\sigma }{2}\right\}$. Furthermore, from the first equality of Equation (32), we derive the following:

(57) $${\left|\dot{e}\left(t\right)\right|}^{2}\leq \frac{2}{\varepsilon^{2}}\left[A^{2}+{\left(\varepsilon K_{0}\right)}^{2}\right]\;{\left|e_{t}\right|}_{s}^{2}.$$

As a result, derived from Equation (42), we obtain the following:

(58) $${\tilde{c}}_{1}{\left\|e_{t}\right\|}_{s}^{2}\leq V_{2}\leq {\tilde{c}}_{2}{\left\|e_{t}\right\|}_{s}^{2},$$

where

(59) $${\tilde{c}}_{1}=P,{\tilde{c}}_{2}=P+\frac{2r^{2}}{\varepsilon^{2}}\left[A^{2}+{\left(\varepsilon K_{0}\right)}^{2}\right].$$

Hence, by taking into account Equations (26), (48) and (56), we derive the following:

(60) $${\dot{V}}_{2}\leq -\frac{{\tilde{c}}_{1}{\tilde{c}}_{3}}{\varepsilon }{\left|e_{t}\right|}_{s}^{2}+c_{4}{\left|e_{t}\right|}_{s}\;\left|\Delta_{2}\left(\cdot \right)\right|.$$

The perturbation term exhibits the characteristic

(61) $$\left|\Delta_{2}\left(\cdot \right)\right|\leq \beta {\left|e_{t}\right|}_{s}.$$

Consequently, we acquire the following:

(62) $${\dot{V}}_{2}\leq -\frac{{\tilde{c}}_{1}{\tilde{c}}_{3}}{\varepsilon }{\left|e_{t}\right|}_{s}^{2}+c_{4}\beta {\left|e_{t}\right|}_{s}^{2}.$$

Differentiating the Lyapunov function W with respect to time gives

(63) $$\dot{W}={\dot{V}}_{1}+{\dot{V}}_{2}.$$

Upon substituting Equations (44) and (62) into Equation (63), we obtain the following:

(64) $$\begin{array}{l} \dot{W}\leq -c_{3}{\left\|X\left(t\right)\right\|}^{2}+l_{1}\left\|X\left(t\right)\right\|\;{\left|e_{t}\right|}_{s}-\frac{{\tilde{c}}_{1}{\tilde{c}}_{3}}{\varepsilon }{\left|e_{t}\right|}_{s}^{2}+c_{4}\beta {\left|e_{t}\right|}_{s}^{2}\\ =-\left[\begin{array}{cc} \left\|X\left(t\right)\right\| & {\left|e_{t}\right|}_{s} \end{array}\right]\;M\;\left[\begin{array}{l} \left\|X\left(t\right)\right\|\\ {\left|e_{t}\right|}_{s} \end{array}\right], \end{array}$$

where

(65) $$M=\left[\begin{array}{cc} c_{3} & -\frac{1}{2}l_{1}\\ -\frac{1}{2}l_{1} & \frac{{\tilde{c}}_{1}{\tilde{c}}_{3}}{\varepsilon }-c_{4}\beta \end{array}\right].$$

As $c_{3},l_{1}$ are positive constants, it is readily verifiable that when M(2,2)>0 and det(M)>0, we have the following:

(66) $$\begin{array}{l} \frac{{\tilde{c}}_{1}{\tilde{c}}_{3}}{\varepsilon }>c_{4}\beta \;\Rightarrow \;\varepsilon <\frac{{\tilde{c}}_{1}{\tilde{c}}_{3}}{c_{4}\beta },\\ c_{3}\frac{{\tilde{c}}_{1}{\tilde{c}}_{3}}{\varepsilon }-c_{3}c_{4}\beta -\frac{1}{4}l_{1}^{2}>0\;\Rightarrow \;\frac{c_{3}\;{\tilde{c}}_{1}{\tilde{c}}_{3}}{\varepsilon }>c_{3}c_{4}\beta +\frac{1}{4}l_{1}^{2}\;\Rightarrow \;\varepsilon <\frac{c_{3}\;{\tilde{c}}_{1}{\tilde{c}}_{3}}{c_{3}c_{4}\beta +\frac{1}{4}l_{1}^{2}}. \end{array}$$

Consequently, if ε is chosen such that $\varepsilon <\min\left\{\frac{{\tilde{c}}_{1}{\tilde{c}}_{3}}{c_{4}\beta },\frac{c_{3}\;{\tilde{c}}_{1}{\tilde{c}}_{3}}{c_{3}c_{4}\beta +\frac{1}{4}l_{1}^{2}}\right\}$, then the matrix M becomes positive definite, leading to the following:

(67) $$\dot{W}\leq -\lambda_{\min}\left(M\right)\;{\left\|Z_{t}\right\|}_{s}^{2},$$

where

(68) $$Z\left(t\right)=\left[\begin{array}{l} X\left(t\right)\\ e\left(t\right) \end{array}\right],Z_{t}\left(t\right)=\left[\begin{array}{c} X\left(t\right)\\ e_{t} \end{array}\right],$$

$\text{and}\;{\left\|z_{t}\right\|}_{s}=\underset{\alpha \in \left[-s,0\right]}{\sup}{\left[{\left\|X\left(\delta \left(t\right)\right)\right\|}^{2}+{\left|e\left(t+\alpha \right)\right|}^{2}\right]}^{\frac{1}{2}}$.

In essence, $\lambda_{\min}\left(M\right)$ is bounded below by a positive constant that does not depend on ε [25]. Integrating Equation (67) results

(69) $$W\left(t\right)\leq W\left(0\right)-\int_{0}^{t}\lambda_{\min}\left(M\right)\;{\left\|Z_{t}\right\|}_{s}^{2}\;dt.$$

Since W(t) is a positive definite function, Equation (69) guarantees the asymptotic stability of the controlled system, meaning that $\lim_{t\to \infty }\;\left\|X\left(t\right)\right\|=0,\lim_{t\to \infty }\;\left\|e_{t}\right\|=0.$

## 5 Results of the Simulation

In this section, the effectiveness of the proposed control strategy for modelling tumour growth, as represented by Equation (1a), (1b) in MATLAB/Simulink, will be demonstrated. Table 1 outlines the relevant system parameters.

**TABLE 1:** Parameters of the system.

Tumour growth rate:	a=0.27[1/day]
Inhibition effect:	b=0.0074[kg/mg/day]
Drug clearance:	c=ln(2)/3.9[1/day]

The initial conditions of system states are defined as $x\left(0\right)=10000\;\left[\;mm^{3}\right]$ and $x\left(0\right)=10000\;\left[\;mm^{3}\right]$.

We simulate the proposed control method for tumour growth as outlined in Equation (1a), (1b). Unlike related studies [25, 26, 27], which rely on online measurement of system states, our approach utilises an observer to estimate tumour growth based on time‐delayed measurements.

The proposed method offers several advantages over similar approaches discussed in the literature as outlined in Table 2. Subsection A compares the results of our method with those presented in ref. [27], while subsection B provides a comparison with the findings from refs. [25, 26].

**TABLE 2:** Comparison of the proposed control method with approaches from references [25, 26, 27].

	Proposed method	Method proposed in [25, 26]	Method proposed in [27]
Taking measurement delays into account:	✓	×	×
Remark: Time delays in measuring data for tumour growth control are critical, as neglecting this factor can destabilise closed‐loop systems and diminish the effectiveness of control measures.			
Taking input delays into account:	✓	×	×
Remark: Time delays in system input for tumour growth control are unavoidable, and neglecting this factor can destabilise closed‐loop systems and reduce the effectiveness of control measures.			
Ensuring the positivity of the system input:	✓	✓	✓
Remark: Considering the positivity of the system input is essential for effective tumour growth control.			
Stability outcomes	Asymptotic ✓	Asymptotic ×	Asymptotic ×
	BIBO ✓	$H_{\infty }$ norm‐based ✓	$H_{2}|H_{\infty }$ norm‐based ✓
Remark: Analysing the stability of an equilibrium point is crucial for understanding the system's long‐term behaviour and ensuring that it responds predictably to disturbances.			
Robustness analysis	Mathematically ✓	Mathematically ×	Mathematically ×
	Simulation ✓	Simulation ✓	Simulation ✓
Remark: Robustness is a key factor in evaluating the effectiveness of controllers.			

### 5.1 Analysis of Comparison Results With [25, 26, 27]

In this section, we commence by simulating the system defined by Equations (1a), (1b) with the parameters outlined in Table 2, employing a method similar to those in references [25, 26, 27]. Lei and Khalil [16] present a methodology for positive input dynamics that involves converting the positive system into a linear framework without considering time delays in system input and output. This transformation enables the design of robust controllers using $H_{\infty }$ norm‐based techniques for the minimal tumour growth model. For simulating the proposed controller, it is assumed that the patient undergoes a scan every 5 days and receives an injection every 2 days. Thus, a delay of s=5days is considered for tumour growth measurement, while a delay of τ=2days is accounted for in the injection input. The performance comparison of the controlled systems with respect to ref. [27] is provided in Table 3.

**TABLE 3:** Comparison of the performance of controlled systems.

	Proposed controller	Ref. [27]
Stability results	Asymptotic	$H_{2}|H_{\infty }$
Robustness to uncertainties	✓	×
Taking into account measurement delay	✓	×
Taking into account input delay	✓	×
Time for tumour cells to converge to zero (days)	15	50
Remaining injection rate (mg/kg/day)	0	10
Maximum percentage of tumour growth compared to the initial tumour size	18%	70%
Maximum rate of injection (mg/kg/day)	15.2	21

**FIGURE 2:**
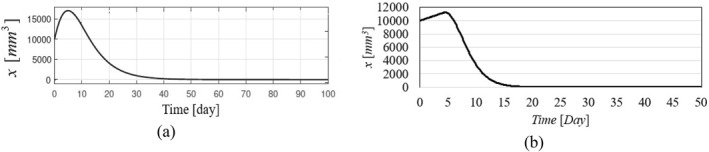
Tumour volume over time: (a) Results using the controller from ref. [27]; (b) Results with the proposed control method.

**FIGURE 3:**
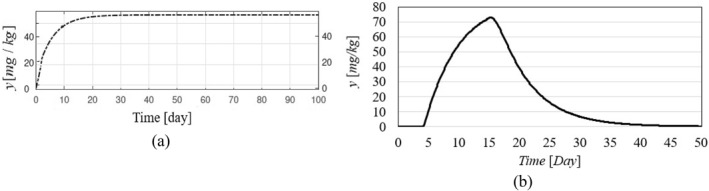
Temporal evolution of the inhibitor level: (a) Results obtained with the controller from ref. [27]; (b) Outcomes achieved using the proposed control method.

Figure 2, 3, 4 through 5 present the time histories of tumour volume, inhibitor concentration, injection rates, and tumour growth predictions for both the proposed control method and the approaches detailed in ref. [27]. Figure 2 illustrates that the proposed method leads to a 20% increase in tumour volume at the onset of treatment, whereas a 70% increase is noted in ref. [27]. This variance is attributed to the initially low inhibitor concentration shown in Figure 3. As the inhibitor concentration rises, the rate of tumour growth diminishes, resulting in more controlled tumour progression.

**FIGURE 4:**
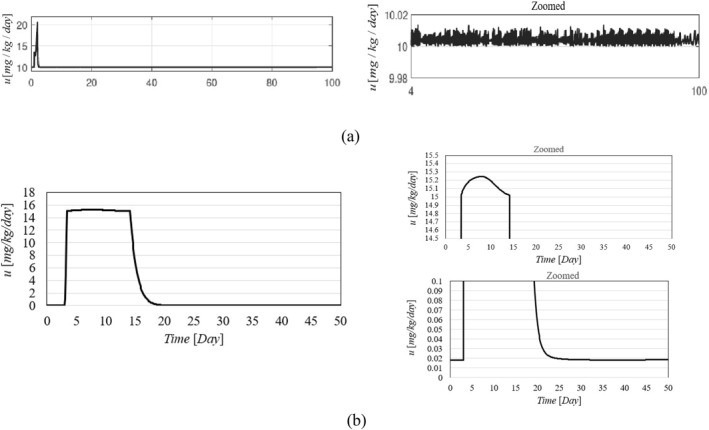
Time history of the injection rate: (a) Controller from ref. [27]; (b) Proposed control method.

**FIGURE 5:**
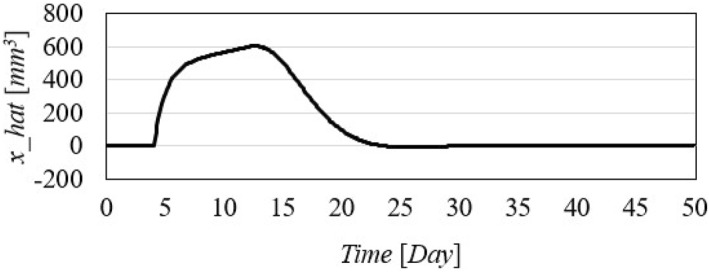
Time history of the predicted state.

Figure 4 illustrates an initial spike in the inhibitor injection rate, designed to suppress tumour growth, which then gradually decreases as the tumour volume reduces. The proposed method shows a high initial injection rate that stabilises after the substantial initial dose. In contrast, the method outlined in ref. [27] exhibits significant fluctuations in the injection rate due to errors in state prediction, potentially diminishing the practical effectiveness of the controller. Additionally, Figure 2 shows that the initial peak in tumour volume is notably reduced with the proposed method compared to the approach in ref. [27]. This improvement is due to the proposed controller's enhanced ability to maintain equilibrium stability, leading to reduced peak tumour volume through precise control adjustments. Figure 5 offers predictions for tumour volume growth.

### 5.2 Robustness Analysis of the System Against Uncertainties

**FIGURE 6:**
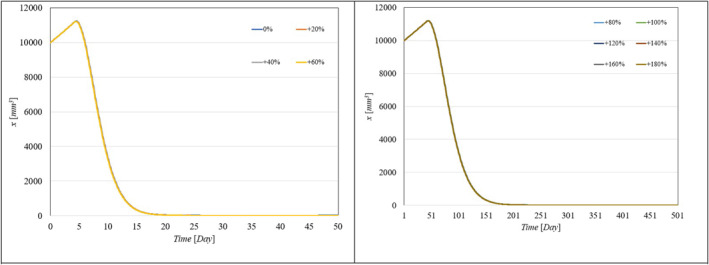
Tumour volume over time with uncertainties in model parameters *a, b, c*.

To showcase the system's robustness in the face of parameter uncertainties, we conducted simulations with the proposed controller under conditions where system parameters varied from 10% to 100% and included different levels of delays in both input and output. Figure 6, 7 through 8 display the resulting time histories for tumour volume, inhibitor levels, and injection rates. Additionally, to further illustrate the controller's robustness against varying time delays, we performed simulations under a range of delays in both input and output. Figure 9, 10 through 11 provide the time histories for tumour volume, inhibitor levels, and injection rates under these conditions.

**FIGURE 7:**
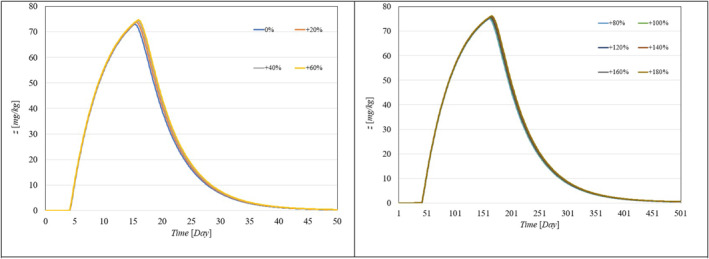
Temporal evolution of the inhibitor level with uncertainties in model parameters *a, b, c*.

**FIGURE 8:**
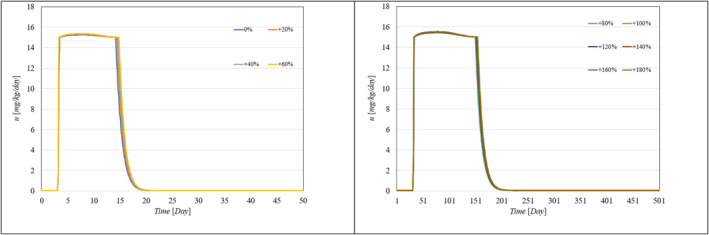
Temporal variation of the injection rate with uncertainties in model parameters *a, b, c*.

**FIGURE 9:**
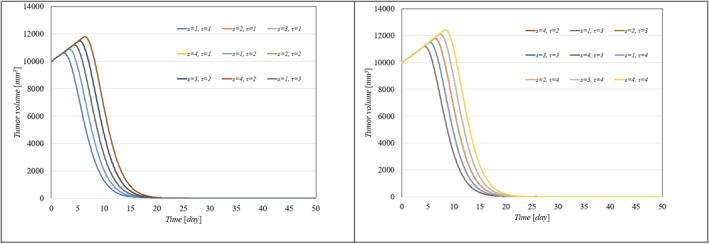
Tumour volume over time with varying time delays in both the system and output, represented by s,τ.

**FIGURE 10:**
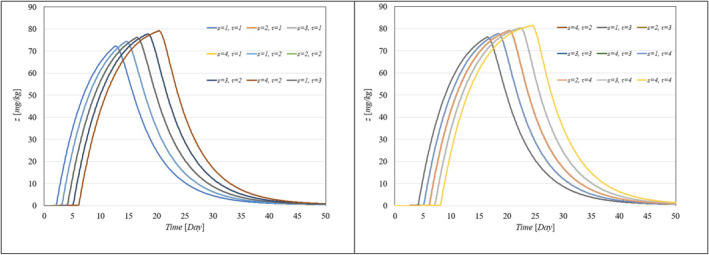
Temporal variation of the inhibitor level with varying time delays in both the system and output, indicated by s,τ.

**FIGURE 11:**
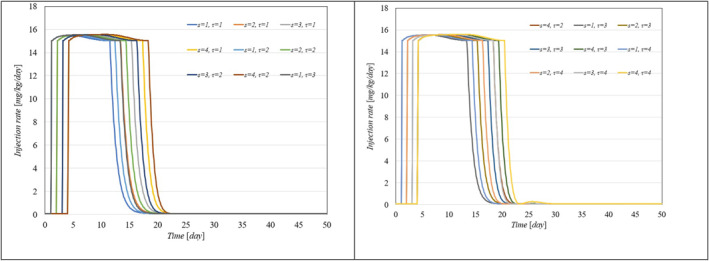
Temporal variation of the injection rate with different time delays in both the system and output, denoted by s,τ.

These results illustrate that the controller consistently upholds its performance, even amidst uncertainties and delays. Furthermore, the findings suggest that as parameter uncertainties increase, a higher injection rate becomes necessary to manage the system effectively.

## 6 Conclusion

This paper presents a novel approach to output feedback control for tumour growth systems, incorporating time delays and positive input. By employing a high‐gain predictor, this method incorporates the predicted and delayed states into the feedback mechanism. The proof of performance recovery highlights the benefits of using a high‐gain predictor‐based bounded controller. This study advances tumour growth control by handling positive input dynamics without requiring system model expansion to ensure input positivity. Consequently, the approach directly controls the actual input, maintaining its bounded nature, which marks a significant improvement over previous methods. The proposed controller operates based solely on tumour volume measurements, with its global asymptotic stability confirmed through the Lyapunov theorem. Additionally, the controller's robustness against uncertainties is validated through the Lyapunov theorem, providing both mathematical and numerical evidence of its BIBO stability. Simulation results highlight the controller's effectiveness and its superiority over previous techniques.

## Author Contributions

All contributions belong to the sole author.

## Conflicts of Interest

The author declares no conflicts of interest.

## Data Availability

The data that support the findings of this study are available from the corresponding author upon reasonable request.

## Appendix A Validating the Robustness of the Closed‐Loop System

In this subsection, we examine the robustness of the closed‐loop system when faced with parametric uncertainties. The nominal values of the system parameters are represented using the notation.

Theorem 4
*For the tumour growth system described by Equation* (1a) *and* (1b), *the controller defined by the following:*

(A1)
$$u\left(t\right)=\bar{c}y^{*}+\bar{b}x^{2},$$

guarantees BIBO stability of the system, even when parametric uncertainties are present.

*Proof.*

By applying a method akin to that detailed in Section 2, the error dynamics of the system under parametric uncertainties can be expressed as follows:

(A2)
$$\dot{x}=\left(a-bk\right)x-bxe,$$

(A3)
$$\dot{e}=-\bar{c}e+\bar{b}x^{2}+r_{d}\left(t\right).$$

Here, $r_{d}\left(t\right)$ represents the deviation of the system parameters from their nominal values, defined as $r_{d}\left(t\right)=-\left(c-\bar{c}\right)e+\left(b-\bar{b}\right)x^{2}$. For all trajectories within the region Ω={X(t)|‖X(t)‖<μ}, the perturbation $r_{d}\left(t\right)$ ibounded, meaning $\left|r_{d}\left(t\right)\right|<\mu_{d}$, where $\mu ,\mu_{d}$ are positive constants.

With this substitution, the Lyapunov function $V_{1}$ can be expressed as $V_{1}=\frac{1}{2}X^{T}X$. Taking into account the system errors with uncertainties, as described by Equations (A2) and (A3), we set μ>0 and $\mu_{d}>0$ such that {‖X(t)‖≤μ}⊂Π and $\left\{\left|r_{d}\left(t\right)\right|\leq \mu_{d}\right\}\subset \Pi_{d}$, where $\Pi \subset R^{n}$ represents the domain containing x=0 and $\Pi_{d}\subset R^{n}$ is the domain encompassing $r_{d}=0$.

Using Equations (A2) and (A3) and employing a method analogous to the one outlined in Section 2, we compute the time derivative of the Lyapunov function as follows:

(A4)
$${\dot{V}}_{1}\leq -\gamma \;{\left\|X\left(t\right)\right\|}^{2}+\left\|X\left(t\right)\right\|\;\left\|r_{d}\left(t\right)\right\|.$$

Let $U=\sqrt{V_{1}\left(x\right)}$. When $V_{1}\left(x\right)\neq 0$, substitute U˙=V˙12V1 and use Equation (A4) to derive the following results:

(A5)
$$\dot{U}\leq -\frac{1}{2}\gamma \;U+\frac{1}{2}\left\|r_{d}\left(t\right)\right\|.$$

When $V_{1}\left(X\right)=0$, it can be confirmed that

(A6)
$$D^{+}U\leq \frac{1}{2}\left\|r_{d}\left(t\right)\right\|.$$

(A7)
$$D^{+}U\leq -\frac{1}{2}\gamma U+\frac{1}{2}\left\|r_{d}\left(t\right)\right\|.$$

By leveraging the Comparison Lemma, we can subsequently ascertain

(A8)
$$U\left(t\right)\leq e^{-\gamma t}\;U\left(0\right)+\frac{1}{2}\int_{0}^{t}e^{-\gamma \left(t-\tau \right)}\left\|r_{d}\left(\omega \right)\right\|\;d\omega .$$

(A9)
$$\left\|X\left(t\right)\right\|\leq \left\|X\left(0\right)\right\|\;e^{-\frac{1}{2}\gamma t}+\frac{1}{2}\int_{0}^{t}e^{-\gamma \left(t-\tau \right)}\left\|r_{d}\left(\omega \right)\right\|\;d\omega .$$

It is straightforward to confirm that

(A10)
$$\left\|X\left(0\right)\right\|\leq \mu ,\underset{0\leq \sigma \leq t}{\sup}\;\left\|r_{d}\left(\sigma \right)\right\|\leq \mu_{d}.$$

This ensures that ‖X(t)‖≤μ, thereby keeping the state x(t) within the domain where the assumptions hold. As a result, the robustness of the system is maintained through the use of a high‐gain controller.
